# Generative Artificial Intelligence for Data Analysis: A Randomised Controlled Trial in a Public Health Research Institute

**DOI:** 10.3389/ijph.2025.1608572

**Published:** 2025-10-01

**Authors:** Tafadzwa Dhokotera, Nandi Joubert, Aline Veillat, Christoph Pimmer, Karin Gross, Marco Waser, Jan Hattendorf, Julia Bohlius

**Affiliations:** ^1^ Department Epidemiology and Public Health, Swiss Tropical and Public Health Institute, Associated Institute of the University of Basel, Basel, Switzerland; ^2^ Swiss Tropical and Public Health Institute, Associated Institute of the University of Basel, Basel, Switzerland; ^3^ Department Education and Training, Swiss Tropical and Public Health Institute, Associated Institute of the University of Basel, Basel, Switzerland

**Keywords:** ChatGPT, data analysis, epidemiology, generative artificial intelligence, higher education

## Abstract

**Objective:**

To assess the competence of students and academic staff to use generative artificial intelligence (GenAI) as a tool in epidemiological data analyses in a randomised controlled trial (RCT).

**Methods:**

We invited postgraduate students and academic staff at the Swiss Tropical and Public Health Institute to the RCT. Participants were randomized to analyse a simulated cross-sectional dataset using ChatGPT’s code interpreter (integrated analysis arm) vs. a statistical software (R/Stata) with ChatGPT as a support tool (distributed analysis arm). The primary outcome was the trial task score (out of 17, using an assessment rubric). Secondary outcome was the time to complete the task.

**Results:**

We invited 338 and randomized 31 participants equally to the two study arms and 30 participants submitted results. Overall, there was no statistically significant difference in mean task scores between the distributed analysis arm (8.5, ±4.6) and the integrated analysis arm (9.4, ±3.8), with a mean difference of 0.93 (p = 0.55). Mean task completion time was significantly shorter in the integrated analysis arm compared to the distributed analysis arm.

**Conclusion:**

While ChatGPT offers advantages, its effective use requires a careful balance of GenAI capabilities and human expertise.

## Introduction

Generative artificial intelligence (GenAI) refers to a class of artificial intelligence (AI) systems designed to create new and original content by learning from large amounts of real-world data, including image and text [1, 2]. Large language models (LLMs) are one of the most prominent types of GenAI, with OpenAI’s ChatGPT (Generative Pre-trained Transformer) being the most widely recognized tool [3]. The advent of ChatGPT and similar GenAI technologies presents both opportunities and challenges for educators. On the one hand, these tools can significantly enhance learning and productivity by improving access to information, providing personalized learning, aiding in generating and refining ideas, writing assistance and feedback, and performing analyses and programming tasks [4, 5]. On the other hand, concerns have been raised about the implications of using these tools for educational purposes, including the risk of hindering students’ development of problem solving and critical thinking techniques, issues of academic integrity, potential misuse, and perpetuating existing biases [4].

Introduced in November 2022 by OpenAI, ChatGPT functions as a multipurpose chatbot and has shown potential in research in the areas of scientific writing, programming and coding, and idea exploration, amongst others [3]. Since its introduction, ChatGPT has undergone significant improvements in accuracy, contextual understanding, functionality, efficiency, and usability, and continue to evolve rapidly. In the domain of data analysis, ChatGPT has the ability to process and analyse data, including tasks such as data extraction, program code generation, statistical modelling, and direct data upload capabilities for more comprehensive analyses [6]. There is a growing concern that GenAI, with its ability to automate tasks and processes, could lead to the disruption of scientific integrity by reducing the need for human expertise, fostering plagiarism and potentially spreading misinformation through the generation of inaccurate or biased content. However, students have largely embraced GenAI, especially ChatGPT, viewing it not as a threat but as a valuable tool for improving productivity, democratising access to complex tasks, and enhancing learning [7]. Therefore, determining the optimal way to integrate GenAI into academic settings to maximise benefits while minimising potential risks remains essential.

Data analysis and interpretation remain some of the most valued and challenging competencies in epidemiology and public health, particularly for students who often enter programs with varying levels of proficiency in statistical programming and analysis [8, 9]. Generative AI tools have the potential to bridge gaps in expertise, enabling students to engage more effectively with complex datasets and analyses. Recent randomised controlled trials (RCTs) showed that the use of GenAI improved the attitudes and understanding of statistics at undergraduate university level [10, 11]. However, another RCT conducted on mathematics students found that those who relied solely on GenAI experienced a 17% decline in outcomes compared to students without AI access, while those who used AI with guidance and teacher input showed no significant difference in performance [12]. Understanding how GenAI impacts students’ abilities to critically analyse epidemiological data is important to ensure they support traditional learning without weakening essential skills.

This highlights the need for a thoughtful approach to incorporating GenAI, particularly ChatGPT, into higher education without disrupting foundational competencies, particularly in fields like epidemiology and public health where data literacy, statistical reasoning, and critical thinking are essential for accurate analysis and evidence-based decision-making.

We therefore conducted a RCT including MSc and PhD students and academic staff (lecturers, supervisors) in the field of epidemiology at a public health institute. We aimed to assess the performance of students and staff when using ChatGPT for epidemiological data analysis—either as a support tool alongside Stata or R (distributed analysis arm), or or as an integrated analytical tool where conversational features are merged with data analysis functions based on ChatGPT’s code interpreter (integrated analysis arm).

From a socio-cognitive point of view, these two study arms go beyond the tool perspective, which would merely distinguish between the use of one vs. two tools to analyse data. Instead, they represent a shift from *distributed cognition* - to what we describe here as *integrated, dialogic cognition*. The consideration of a cognitive system - beyond the mind of an individual - that includes interactions with the environment, physical and digital artefacts, and other people has been prominently coined as distributed cognition [13]. The concept emphasises that knowledge and problem solving are distributed across social (people) and physical structures (e.g., tools) of a system. This is how the first arm of the study can be described: users interact with two (distributed) tools, e.g., Stata and ChatGPT. With this approach, ChatGPT can be conceived as a separate, supportive agent [14] that demonstrates human-like actions, with its prosocial behaviours shaped by the users’ utterances [15]. The entire system, i.e., the interplay between the agents and the use of the tool presents a classic distributed cognitive system. In the second arm, where study participants use ChatGPT’s code interpreter, cognition unfolds dialogically in one, much more tightly integrated system, through conversational turns between a human actor and a machine, a digital cognitive agent, bound to one virtual (tool) environment. The knowledge product is co-constructed in a confined space through an open-ended engagement.

## Methods

### Trial Design, Setting and Participants

We conducted a RCT to evaluate the competence of postgraduate students and academic staff in analysing epidemiological data using GenAI, specifically ChatGPT. The RCT consisted of two study arms: the *distributed analysis* arm allowed participants to use statistical analysis software (Stata or R) with ChatGPT support, whereas participants in the *integrated analysis* arm solely used the integrated data upload and analysis function within ChatGPT. Both arms were allowed to consult the internet for additional assistance.

The research team leading the study included senior epidemiologists, a senior statistician, a postdoctoral researcher and a PhD student. The Swiss Tropical and Public Health Institute (Swiss TPH) is a multidisciplinary public health research and academic institute. We invited all Swiss TPH postgraduate students (MSc or PhD students) as well as academic staff with teaching assignments at Swiss TPH to participate in the study. MSc participants were either studying a Master of Science in Epidemiology or a Master of Science in Infection Biology. To be eligible, participants were required to have completed key courses in epidemiology and statistics or demonstrate equivalent skills through professional experience, ensuring they had the necessary competence to complete the study task.

### Randomisation and Allocation Concealment

Participants were randomised into the distributed and integrated analysis arms stratified by their role at Swiss TPH, categorized as either academic staff or student (MSc and PhD). This stratified randomisation ensured that both groups had a balanced representation of participants based on their professional roles. Allocation concealment was achieved by providing participants with opaque sealed envelopes that indicated their assigned group. The two independent task reviewers were not informed about the group assignments of the participants, thus allowing for an unbiased evaluation of the task outcomes. However, it was noted that the task reviewers could infer some group assignments based on the type of outputs presented and the comments left by participants. Participants copied and pasted the outputs of their analysis from the respective data analysis programs which, in some cases, revealed the arm they were in. It was not possible to blind the participants due to the study design.

### Data Analysis and Outcome Measures

Before the trial, we invited all potential trial participants to a 1-h information session on the use of ChatGPT with integrated data upload and analysis function to ensure they had a basic understanding of its functionalities, capabilities, and limitations. All participants were provided with a ChatGPT subscription from the information session until the trial date (1 month). This served as both an incentive for participation and as an opportunity to encourage engagement with ChatGPT before the trial, allowing participants to familiarise themselves with its functionalities and gain confidence in using it for data analysis and interpretation.

On the day of the trial, all participants were given the same 45-min task, which involved conducting a cross-sectional analysis of a simulated epidemiological dataset. The task, developed by a senior statistician, reviewed by a second senior statistician and piloted by a postdoctoral staff member. Participant solutions were evaluated using a 22-point checklist, with each item assessed as either correct or incorrect. Depending on the relevance of the item, correct responses were awarded 0.5 or 1 point. For the primary outcome, all the points were added together to give a score ranging from 0 to a maximum of 17. The assessment focused on three main assessment areas: descriptive analysis, inferential statistics, and data/result visualisation. For the descriptive analysis, participants were evaluated on how they presented a 2 × 2 contingency table. For the inferential analysis, evaluation was based on choice of statistical model, handling of confounders, colliders, nuisance variables and categorical variables, presentation of results and correct result interpretation. In addition, participants got extra points for statistical model diagnostics. Participants were awarded data visualisation points for informative plots with legends that accurately described the figure.

The primary outcome was the score obtained in the trial task out of a maximum of 17 points. For evaluation of the task, two reviewers graded each task using a predefined assessment rubric. Agreement between both raters were high: the median absolute difference between the scores was 0.5 points (IQR: 0–1). Each difference was reviewed and discussed and finally rated on a consensus. In cases where a consensus could not be reached, the average of the scores was taken to ensure a fair and balanced evaluation. The trial task was evaluated across three domains: descriptive, inferential and data visualisation. Secondary outcomes included the time taken to complete the individual trial tasks and a qualitative analysis of code mistakes as well as responses from open-end questions, where participants described their interactions with ChatGPT.

### Data Collection, Management and Statistical Analysis

We collected demographic information (age and gender) and educational and professional background information (highest educational degree, current professional role, statistical programming and ChatGPT experience) from participants. Statistical programming and ChatGPT experience were self-rated into three groups, beginner, intermediate, and advanced. Participants were assigned an overall programming level based on their individual self-reported competence in Stata, R, and Python. Although the trial required participants to use Stata or R, Python was included in the assessment because it is the primary language underlying ChatGPT’s data analysis capabilities and may influence participants’ ability to interact effectively with the tool. Those who rated themselves as “Advanced” in at least one language were classified as “Advanced,” while those with at least one “Intermediate” rating were classified as “Intermediate,” and the rest as “Beginner.” In addition to the task scores, we collected statistical codes, analysis outputs and comments from users on their experience with using ChatGPT or traditional statistical software for the analysis of the study task.

We estimated the mean score with the corresponding standard deviation and the mean difference in scores in the two trial arms. We evaluated whether scores differed by participants’ educational and professional level, i.e., students versus academic staff, age, gender, attendance of introductory ChatGPT session and statistical as well as ChatGPT experience. We evaluated factors influencing the differences in test scores between arms using linear regression. Each participant was required to: (1) select a statistical approach, (2) identify and interpret the correct estimate, (3) report a confidence interval, (4) choose covariates, and (5) incorporate categorical variables. We recorded the proportion completing each stage to evaluate performance decline. Using qualitative methods, we investigated statistical coding mistakes and participants’ reported experiences with using ChatGPT for statistical analysis and visualization tasks. The responses were analysed to find themes which were categorized as: strengths, challenges and opportunities.

### Sample Size

**FIGURE 1:**
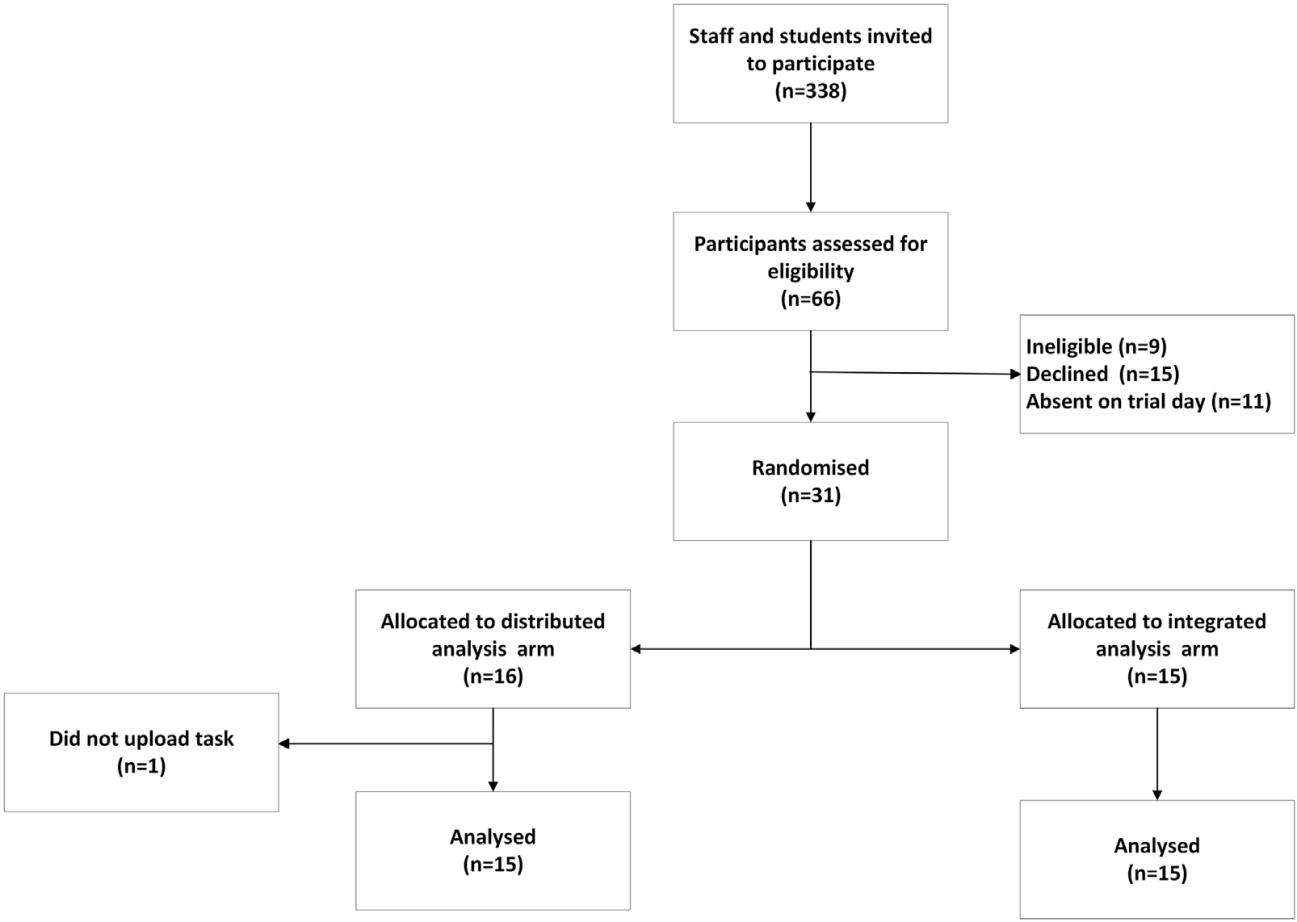
Study flow diagram (Generative artificial intelligence for data analysis: A randomised controlled trial in a public health research institute, Basel, Switzerland, 2024).

In our initial sample size calculation, we aimed to recruit at least 36 participants per arm to detect a difference in scores of 2, with 80% power at the 95% confidence level, assuming a standard deviation of 3. We invited 338 individuals to participate, of these 66 individuals completed the registration form with 42 meeting the eligibility criteria (Figure 1). A final of 31 participants attend the trial on the day of the task, with 16 participants randomised to the distributed analysis arm and 15 participants to the integrated AI arm. One participant did not submit their task for evaluation and was therefore excluded from the outcome analysis. Whilst their demographic data were included in Table 1, which reports the baseline characteristics of all enrolled participants, outcome analyses were conducted on a complete-case basis with 30 participants.

**TABLE 1:** Participant characteristics by study arm (Generative artificial intelligence for data analysis: A randomised controlled trial in a public health research institute, Basel, Switzerland, 2024).

Characteristics	Distributed analysis arm (n = 16)	Integrated analysis arm (n = 15)
	N (%)	N (%)
Role		
Student	10 (62.5)	9 (60.0)
Staff/other	6 (37.5)	6 (40.0)
Age [years]		
>35	7 (43.8)	3 (23.1)
≤35	9 (56.2)	10 (76.9)
*Prefer to not declare*	*0*	*1*
Gender		
Woman	11 (68.8)	9 (66.7)
Man	5 (31.3)	6 (33.3)
Attended ChatGPT information session		
Yes	11 (73.3)	8 (57.1)
No	4 (26.7)	6 (42.9)
*Prefer to not declare*	*1*	*0*
Statistical programming experience		
Advanced	4 (25.0)	7 (46.7)
Intermediate	9 (56.3)	8 (53.3)
Beginner	3 (18.8)	0 (0.0)
ChatGPT experience		
Advanced	1 (6.25)	0 (0.0)
Intermediate	9 (56.3)	11 (73.3)
Beginner	6 (37.5)	4 (26.7)

### Changes to the Study Protocol

We initially had conceptualized a third trial arm with participants analysing data using only Stata or R without ChatGPT or internet support. However, because of the low number of trial participants, we had to remove one trial arm. We decided to remove the one that was least representative of standard data analysis practices, where the standard approach now involves using statistical programs and support from the internet or GenAI. A larger sample size would also have allowed us to evaluate whether scores differed by participants’ characteristics, specifically: age, gender, and experience with statistical programming and ChatGPT. In a post-hoc analysis we assessed participants’ ability to conduct epidemiological analyses without making serious mistakes in both study arms, where serious was defined as mistakes leading to wrong results.

### Ethical Statement

This project was reviewed by the Ethics Committee Northwest and Central Switzerland (EKNZ). The EKNZ clarified that the project does not fall under the scope of the Human Research Act (HRA Art. 2 Abs. 1), as it is not defined as research concerning human diseases or the structure and function of the human body. Consequently, no ethical authorization was required for this study. All participants provided informed consent at the time of registration, agreeing to participate in the study and to the use of their data for analysis. No identifiable personal data were collected.

## Results

### Quantitative Analysis

We invited 338 individuals to participate, and 66 individuals completed the registration form with 42 people eligible to participate (Figure 1). A final of 31 participants were included in the study, with 16 randomized to the distributed analysis arm and 15 participants to the integrated AI arm. Compared to participants in the distributed analysis, those in the integrated analysis arm were younger, with 76.9% aged 35 or less (Table 1). There was a higher proportion of postgraduate students (vs. academic staff) and women in both arms. Attendance of the ChatGPT information session was higher in the distributed analysis arm (73.3%) compared to the integrated analysis arm (57.1%). In terms of statistical programming experience, a higher percentage of participants in the integrated analysis arm self-rated as Advanced (46.7% compared to 25.0% in the distributed analysis arm). Overall, there was a non-significant difference in the mean task scores between the distributed analysis arm (8.5 ± 4.6 standard deviations (SD)) and the integrated analysis arm (9.4 ± 3.8 SD), with a mean difference of 0.93 (p = 0.55). Participants in the integrated analysis arm completed the task significantly faster than those in the distributed analysis arm (mean time 38 min versus 45 min, p = 0.002) (Table 2).

**TABLE 2:** Mean task score by randomised controlled trial arm (Generative artificial intelligence for data analysis: A randomised controlled trial in a public health research institute, Basel, Switzerland, 2024).

Characteristics	Distributed analysis arm	Integrated analysis arm	Mean difference	Univariable analyses		Multivariable analyses	
	N = 15	N = 15		Estimate	95% CI	Estimate	95% CI
	Mean (S.D)	Mean (S.D)					
Overall task score	8.50 (1.20)	9.43 (3.80)	0.93		−2.24, 4.11	−0.26	−3.16, 2.64
Stratum						
Staff/other	9.83 (4.73)	10.3 (4.08)	0.42	Reference			
Student	7.61 (4.64)	8.89 (3.75)	1.28	−1.79	−4.98, 1.39		
Gender						
Man	8.20 (4.79)	7.5 (4.27)	0.70	Reference			
Woman	8.65 (4.82)	10.4 (3.36)	1.75	1.68	−1.65, 5.00		
Age [years]						
>35	9.08 (4.38)	10.8 (3.25)	1.75	Reference			
≤35	8.11 (5.03)	8.64 (3.84)	0.53	−1.27	−4.72, 2.19		
Attended ChatGPT information session						
No	12.7 (1.89)	11.8 (1.13)	0.84	Reference		Reference	
Yes	7.86 (4.56)	7.83 (4.16)	0.03	−4.26	−7.27, −1.25	−4.32	−7.45, 1.18
Statistical programming experience						
Advanced	9.88 (5.50)	9.86 (3.88)	0.02	Reference			
Intermediate	8.00 (4.48)	9.06 (3.96)	1.06	−1.42	−4.68, 1.85		
ChatGPT experience						
Intermediate	10.2 (3.88)	9.32 (4.06)	0.83	Reference			
Beginner	5.20 (4.59)	9.75 (3.50)	4.55	−2.49	−5.84, 0.86		

The *distributed analysis* arm allowed participants to use statistical analysis software (Stata or R) with ChatGPT, support, whereas participants in the *integrated analysis* arm solely used the integrated data upload and analysis function within ChatGPT.

**FIGURE 2:**
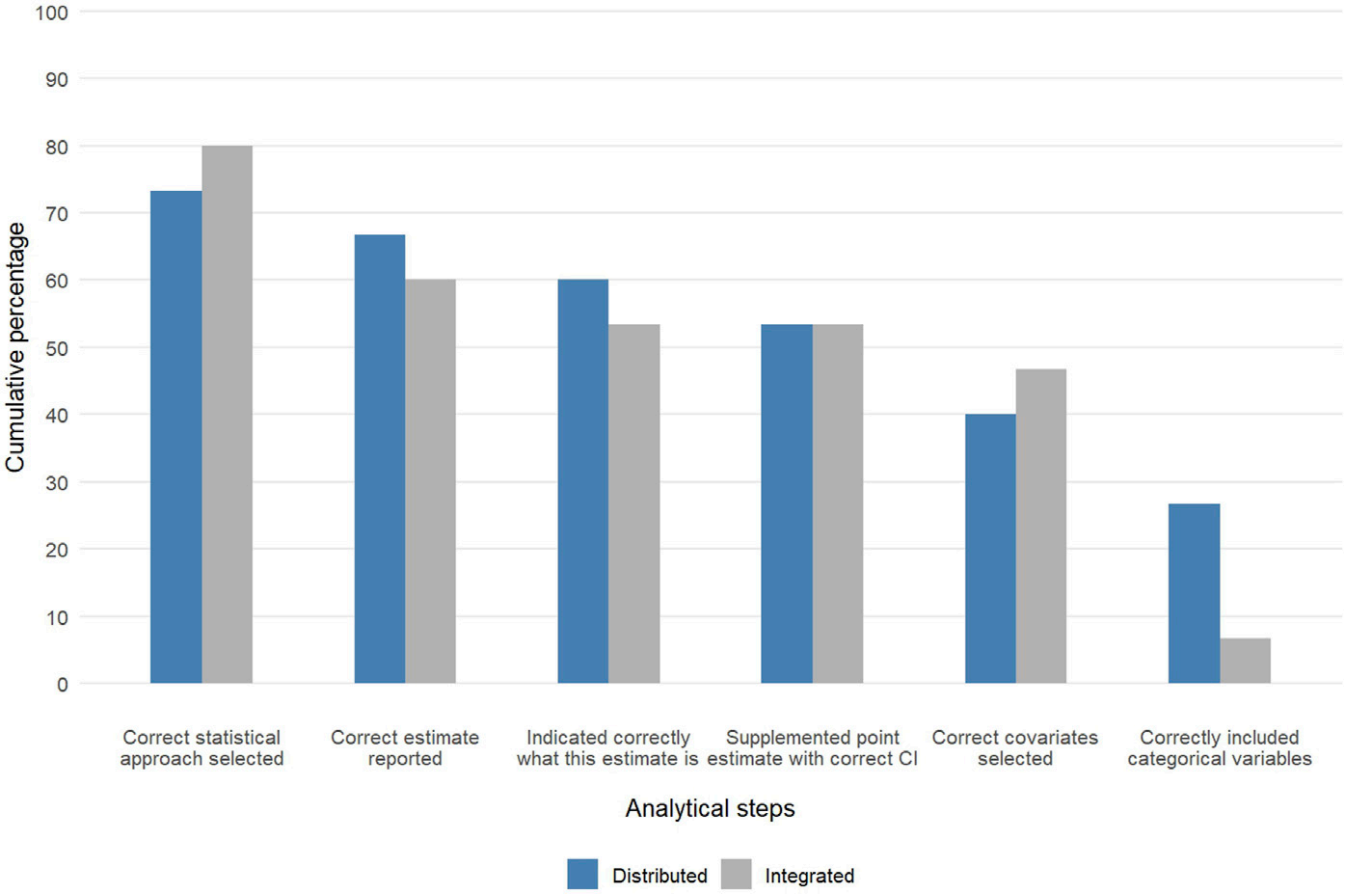
Stepwise performance decline in epidemiological data analysis across study arms (Generative artificial intelligence for data analysis: A randomised controlled trial in a public health research institute, Basel, Switzerland, 2024).

**FIGURE 3:**
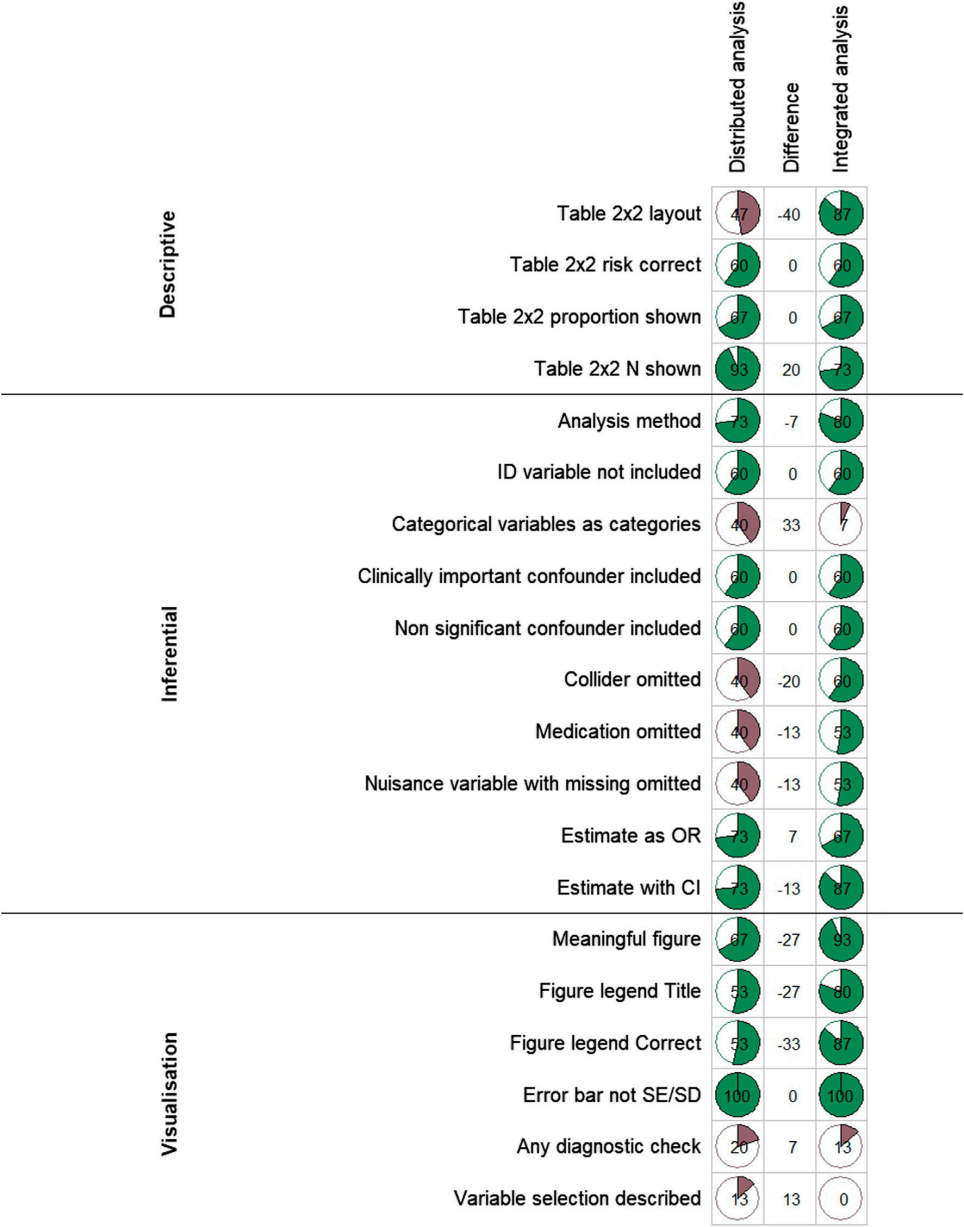
Comparison of performance across different statistical analysis steps between the ChatGPT analyst- and Distributed analysis arms. (Generative artificial intelligence for data analysis: A randomised controlled trial in a public health research institute, Basel, Switzerland, 2024). Each row represents a specific analysis task, with the percentage of correct responses displayed in pie charts. In green are scores above 50% whilst in red are scores below 50%. The middle column indicates performance differences between the two arms.

Figure 2 illustrates the cumulative decline in the number of participants at critical stages of the analysis process for the distributed analysis arm and the integrated analysis arm. Compared to 6.7% in the integrated analysis arm 26% of those in the distributed analysis arm were able to analyse the data without making serious mistakes. Figure 3 presents in detail the performance of participants across analytical steps by trial arm. In the descriptive section, a higher proportion of participants in the integrated analysis arm correctly presented a 2 × 2 contingency table, representing the ability to accurately organize and interpret categorical data (Figure 3). Regarding inferential statistics, a greater proportion of participants in the distributed analysis arm selected the correct model, evaluated categorical and confounding variables, and presented the results accurately compared to the ChatGPT analyst arm (Figure 3). The visualizations for data and results produced in the ChatGPT analyst arm were more meaningful and correctly labelled compared to those in the distributed analysis arm. From the linear regression analyses, having attended the ChatGPT introductory session was the only factor associated with a difference in scores between the ChatGPT analyst and distributed analysis arms. Upon stratifying the analysis by academic staff and postgraduate students, the effect was only statistically significant for students [−4.56; 95% confidence interval (CI): −8.76, −0.36] (Supplementary Table S1). These findings were unexpected and should be interpreted with caution.

### Qualitative Analysis

#### Strengths

Participants’ responses to open ended questions revealed varied experiences with using ChatGPT for statistical analysis and visualization tasks. Many participants highlighted the ease of using ChatGPT, noting positive interactions, particularly for simple descriptive analysis and logistic regression tasks. One of the main strengths observed was the speed at which participants completed the task which was consistent with the quantitative analysis. Participants generally found ChatGPT to be a time-saving tool compared to traditional methods. Additionally, participants in the distributed analysis arm noted that it helped optimize the time they spent on the task. In particular, a participant stated, *“Without the use of ChatGPT … , I would not have been able to do this in the given time frame”.* Another participant stated, *“I added the context of this analysis and the questions asked in the trial to ChatGPT […]. After uploading the data in it, the results came out in a very short time.”*

#### Challenges

Participants encountered several challenges while using ChatGPT. Several participants mentioned technical issues when uploading data files, particularly in relation to browser compatibility. Additionally, visualization tasks were frequently problematic, with participants reporting missing titles and captions in the generated plots and receiving incorrect initial suggestions for plot types. Some participants reported that ChatGPT provided correct interpretations of the results, but there were instances where results were incorrectly interpreted as odds ratios when they were actually un-exponentiated coefficients. One participant in the ChatGPT analyst group mentioned that, “*ChatGPT reported the un-exponentiated coefficient for smoking, but stated it was an Odds Ratio. The estimate was lower than 1, but ChatGPT said that it is above 1 and is therefore associated with an increase in the odds of developing COPD.”*

#### Opportunities

Despite the observed challenges associated with using ChatGPT, several opportunities and effective uses were identified. From the participants’ comments we observed that, some knowledge of statistical analysis or statistical programming led to more precise prompts when using ChatGPT and critical evaluation of generated outputs. This knowledge enabled them to adjust their prompts effectively to achieve the desired result, highlighting the value of combining domain knowledge with advanced analytical tools for optimal performance. One participant mentioned that, “*I then had to tell ChatGPT [it] myself which graph to use and how to graph the data. I had to tell it to add CIs manually…. With visualization, it faced a few hiccups and needed some guidance but once you tell it what to do it was fine*.”

## Discussion

This study assessed the performance of postgraduate students and academic staff to analyse an epidemiological cross-sectional data set with traditional statistical software (Stata or R) and ChatGPT as collaborative tool versus using ChatGPT’s integrated data upload and analysis function exclusively. We found no significant difference in the overall mean trial score between the study arms. We further observed that participants in the *ChatGPT Analyst* arm were more time efficient with reference to completing the task compared to participants in the distributed analysis arm. In addition, from participants’ written comments we observed that advanced analytical and statistical programming knowledge was beneficial in recognising and correcting ChatGPT’s outputs when performing epidemiological analysis.

To the best of our knowledge, this is the first RCT to assess ChatGPT as an analytical collaborator for epidemiological data analysis. By including participants with varying levels of statistical and data analysis expertise, we aimed to capture how different users interact with ChatGPT, providing a more comprehensive understanding of its usefulness. In addition, the trial task was administered under exam conditions, directly assessing participants’ competence to perform the analysis. However, our study also has several limitations. This was an exploratory study with a small sample size and insufficient power to detect small to moderate effects. As such, the results should be interpreted cautiously. Although we blinded the task reviewers to minimize bias in evaluating the outcomes, the task reviewers could infer the participants’ group, in some cases, based on the outputs as they were copied and pasted from the respective programmes. To mitigate this risk, we implemented independent evaluations of the tasks, followed by structured discussions and moderation to ensure consistency and objectivity in assessments. The 45-min task may also not accurately reflect the application of ChatGPT in longer-term epidemiological projects potentially limiting the applicability of our results to real-world scenarios. In addition, we used a simulated dataset, which allowed for data uploads to ChatGPT - a process that may differ in real-world scenarios where ethical considerations and data privacy regulations might restrict data uploads. Technical issues encountered by participants could have affected their performance, introducing variability unrelated to the capabilities of ChatGPT or the participant. Due to the low participation rate, we had to exclude a control arm, preventing comparisons with traditional data analysis methods where GenAI was neither used as a collaborator nor as a standalone analysis tool. Finally, given the rapid advancement of ChatGPT and other LLMs, the challenges identified in this study may evolve or become less relevant by the time of publication.

Our study found that participants in the integrated analysis arm took less time to complete the required tasks than those in the distributed analysis arm. Conceptually, the integrated cognitive system outperformed the distributed cognitive system in terms of speed. The speed gains may be explained by the additional effort required to manage and translate between two different programmes, i.e., Stata/R vs. and GPT, in the distributed analysis arm, which was not needed in the integrated analysis arm, where the statistical results stemmed directly from the conversation between human and digital agent. The level of cognitive integration (vs. distribution) did not affect the overall quality of the task output itself, i.e., the task scores. The quality of the output may depend much more on the user’s competence to develop a critical and reflective stance in conversation with the digital agent [16].

Although our study did not find significant differences in the quality of analysis between using traditional statistical software and those using ChatGPT, an unexpected finding was that attendance of the ChatGPT information session was associated with lower task performance, particularly among students. While statistically significant, this result should be interpreted cautiously. The study was not designed to assess the causal effect of the training session, and we suspect this may reflect unmeasured residual confounding. It is also possible that students attending the session may have relied more on GenAI without fully verifying outputs, though we lack data to confirm this. As such, we do not interpret the information session itself as the cause of poorer performance but instead highlight the need for further research to better understand if and how brief AI training interventions influence user behaviour and learning outcomes.

In our study, participants frequently noted the need for multiple guided prompts to generate accurate graphs and meaningful results with ChatGPT. We also observed that ChatGPT produced errors, consistent with previous research assessing the statistical problem-solving capabilities of different ChatGPT versions. A non-randomized study found that certain ChatGPT models performed poorly on statistical problems such as chi-square tests, one-way ANOVA, and sample size calculations [17]. Similarly, a study evaluating ChatGPT’s use in pharmacokinetic analyses reported that while the ChatGPT correctly provided mathematical formulas, its accuracy dropped to 50% for calculations involving exponential arithmetic [18]. In line with these findings, we observed that ChatGPT misinterpreted un-exponentiated coefficients as odds ratios or provided exponentiated coefficients together with un-exponentiated confidence intervals. This highlights a broader limitation of the version of ChatGPT used in this RCT: when handling statistical outputs, it generates text one token at a time, predicting each token based only on the preceding context rather than following a pre-planned structure. While newer models have improved context awareness, this step-by-step generation process still limits the model’s ability to perform complex reasoning or solve problems that require structured, multi-step planning, as it does not pre-conceptualise the entire response before generating it. Our study included postgraduate students and academic staff with varied statistical experience and showed that participants with more knowledge of statistical programming or analysis were better positioned to solve factual errors produced by ChatGPT. This is in line with previous studies, which showed that users who had expertise in the subject matter could address these limitations [19, 20].

From our study results, educators should carefully consider how to incorporate GenAI into the curricula to optimize students’ data analysis competence. Generative AI has been shown to make statistical learning more accessible to students with varying levels of expertise, improving their attitudes towards statistics [10] and learner outcomes in various studies [12, 21]. Our results also suggests that GenAI can be used to complement human effort in data analysis. While GenAI excels in result presentation including data visualisation, humans contribute with critical methodological reasoning and evaluation, and can bring context to the results. In particular, careful evaluation of ChatGPT outputs and multiple prompts by participants was needed to refine the outputs provided by ChatGPT. As per the UNESCO’s report on AI competency for students, the education system must equip learners with the competencies required to use AI proactively [22]. This includes a human-centred critical approach when engaging with AI, while also understanding its ethical implications and the technical aspects of the AI tools they utilise [23]. To address LLM misinformation, hybrid AI models such as Retrieval-Augmented Generation are emerging to improve accuracy of AI in educational settings by combining AI responses with real-time extraction of verified academic sources [24, 25].

While this study provides valuable insights into the potential and challenges of incorporating generative AI into epidemiological and public health research and education, several questions remain unanswered. Integrated analyses are likely to become a key pathway in future human-computer interaction, where software programs will move beyond traditional navigation features and users will draw on conversational AI to operate a programme. Research is needed to determine the long-term effects of AI-assisted learning on learning and critical thinking [26]. Further studies are needed to identify the most effective training approaches for lecturers to enhance students’ learning and develop their data analysis competence using GenAI. Additionally, a larger study would provide more understanding on how different levels of expertise and varying educational backgrounds affect interactions with GenAI. Finally, exploring the ethical implications, including issues of data privacy, AI bias, and the potential for dependency on AI in education, remains an important area for future research.

### Conclusion

This trial demonstrates the potential of ChatGPT as a powerful tool for collaboration in epidemiological data analysis. While ChatGPT in particular, offers significant advantages in result presentation, humans can bring in critical thinking and contextualisation. Balancing the use of GenAI with traditional learning methods is essential to ensure that it enhances, rather than hinders, the journey to becoming an expert.

## Data Availability

Anonymized summary data that support the findings of this study are available from the corresponding author upon reasonable request.
