# Supplementary material for: Generative Artificial Intelligence for Data Analysis: A Randomised Controlled Trial in a Public Health Research Institute
Source: Int J Public Health. 2025 Oct 1;70:1608572. doi: 10.3389/ijph.2025.1608572 (PMC12521002; doi:10.3389/ijph.2025.1608572)
Supplement: Supplementary file 1 [file Table1.docx]

**Supplementary table 1: Factors associated with trial score, stratified by role**

|  | **Academic staff/ other** | | **Students** | |
| --- | --- | --- | --- | --- |
|  | **Estimate** | **95% CI** | **Estimate** | **95% CI** |
| **Trial arm** | | | | |
| Distributed analysis | Reference | | Reference | |
| ChatGPT analyst | 0.93 | -6.76, 4.91 | 0.32 | -3.51, 4.15 |
| **Attended ChatGPT information session** | | | | |
| No | Reference | | Reference | |
| Yes | -4.04 | -10.23, 2.15 | -4.56 | -8.76, -0.36 |
